# *“Day or night, no matter what, I will go”*: Women’s perspectives on challenges with follow-up care after cervical cancer screening in Iquitos, Peru: a qualitative study

**DOI:** 10.1186/s12905-023-02414-z

**Published:** 2023-05-31

**Authors:** Rachel M. Morse, Magdalena Jurczuk, Joanna Brown, Lita E. Carrillo Jara, Graciela Meza, E. Jennifer Ríos López, J. Kathleen Tracy, Patti E. Gravitt, Valerie A. Paz-Soldan, the Proyecto Precancer Study Group, Meda Del Carpio-Morgan, Henrry Daza Grandez, Magaly Figueredo Escudero, Esther Y. Garcia Satalay, Sarah D. Gilman, Karina Gonzales Díaz, José Jerónimo, Alcedo Jorges, Anna Kohler-Smith, Margaret Kosek, Gabriela Ladrón de Guevarra, Daniel Lenin de Cuadro, Renso Lopez Liñán, Andrea Matos Orbegozo, Jaime Marín, Helen E. Noble, Victor A. Palacios, Reyles Ríos Reátegui, Karina Román, Anne F. Rositch, Carlos Santos-Ortiz, Hermann F. Silva Delgado, Sandra Soto, Nolberto Tangoa, Javier Vásquez Vásquez, Giannina Vásquez del Aguila, Karen Zevallos

**Affiliations:** 1 Department of Global Community Health and Behavioral Sciences, School of Public Health and Tropical Medicine, Tulane University, New Orleans, LA USA; 2 Asociación Benéfica PRISMA, Lima, Peru; 3 Department of Cancer Control and Prevention, Dirección Regional de Salud de Loreto, Iquitos, Loreto, Peru; 4 Facultad de Medicina Humana, Universidad Nacional de La Amazonia Peruana, Iquitos, Peru; 5 Department of Epidemiology and Public Health, University of Maryland School of Medicine, Baltimore, MD USA; 6 Department of Tropical Medicine, School of Public Health and Tropical Medicine, Tulane University, New Orleans, LA USA

**Keywords:** Lost to follow-up, Cervical Cancer, Screening

## Abstract

**Background:**

The study’s objective was to explore the factors associated with loss to follow-up among women with abnormal cervical cancer screening results in Iquitos, Peru from women’s perspectives.

**Methods:**

In-depth interviews were conducted with 20 screen-positive women who were referred for follow-up care but for whom evidence of follow-up was not found. Interview transcripts were thematically analyzed inductively, and the codes were then categorized using the Health Care Access Barriers Model for presentation of results.

**Results:**

All interviewed women were highly motivated to complete the continuum of care but faced numerous barriers along the way, including cognitive barriers such as a lack of knowledge about cervical cancer and poor communication from health professionals regarding the process, structural barriers such as challenges with scheduling appointments and unavailability of providers, and financial barriers including out-of-pocket payments and costs related to travel or missing days of work. With no information system tracking the continuum of care, we found fragmentation between primary and hospital-level care, and often, registration of women’s follow-up care was missing altogether, preventing women from being able to receive proper care and providers from ensuring that women receive care and treatment as needed.

**Conclusions:**

The challenges elucidated demonstrate the complexity of implementing a successful cervical cancer prevention program and indicate a need for any such program to consider the perspectives of women to improve follow-up after a positive screening test.

## Background

Cervical cancer is the fourth most common form of cancer among women worldwide, and in 2020, cervical cancer accounted for an estimated 7.7% of cancer-related deaths among women [1]. In Loreto – the largest state within the Peruvian Amazon rainforest – cervical cancer is the leading cause of cancer-related deaths among women, with a mortality rate 2.6 times higher than the global average and 2.3 times the national average [2–5]. Unless new strategies are implemented, cervical cancer deaths will rise significantly in coming years [6].

Cervical cancer is also highly preventable. Vaccination against human papillomavirus (HPV) – the primary cause of cervical cancer – is an effective way to prevent HPV infection [7]. For those with HPV, a variety of early detection and treatment (EDT) programs can prevent development of cervical cancer [6]. For an EDT program to be effective, multiple components of a complex system must run efficiently, including screening, follow-up, and treatment for those who need it.

Cervical cancer prevention has been a national priority for the Peruvian Ministry of Health (MINSA) since 1998. HPV vaccination and EDT programs have been in place for over a decade with free services available through the public health system [8, 9]. Despite this, the cervical cancer mortality rate in Peru has remained stagnant [5], due in part to high numbers of screen-positive women who are lost to follow-up. Women who are lost to follow-up do not reach an appropriate “endpoint” in their continuum of care. This “endpoint” can be a negative confirmatory test (such as a repeat screening test or colposcopy) or successfully completed treatment [5, 10].

In Latin America, high rates of loss to follow-up are associated with various systemic factors, including inadequate health system infrastructure and insufficient staffing. These systemic factors culminate in difficulties with scheduling care, delays in informing patients of their results, delays throughout the referral process, and poor communication from healthcare staff [11–15]. Moreover, individual factors such as caregiving responsibilities for children or other family members, as well as fears of a cancer diagnosis, death, and loss of reproductive function, were commonly reported reasons for poor adherence to follow-up care in Latin America [11, 12, 14, 16]. Other frequently reported obstacles include high costs of care and high costs of transportation to the care facility, as well as opportunity costs due to missing work to attend a follow-up appointment [12–15, 17].

In Latin America, loss to follow-up rates among screen-positive women have been found to be between 18.3% and 56% [8, 11–14, 16, 18], and a rural region of Peru reported rates as high as 75% in 2003 [17]. This study is part of a larger implementation study – the Proyecto Precancer [19] – based in the Loreto region of Peru, which confirmed these alarmingly high rates of loss to follow-up in Iquitos (capital of Loreto) with routinely collected monitoring and evaluation data. Between January 2018 and June 2019, our records indicated a loss to follow-up rate of 69.8% (120/172) among women who tested positive following a visual inspection with acetic acid (VIA) test (one of three types of screening tests offered) in the Micro Red Iquitos-Sur (MRIS) health network of Loreto [20].

Previous studies on reasons for loss to follow-up following cervical cancer screening have concluded that it is critical to address barriers to women’s continuum of care in order to have an effective EDT program [6, 18]. This study aims to ensure that women have a voice in this process. The objective of this study is to examine the experiences and challenges associated with obtaining follow-up after an abnormal cervical cancer screening result from women’s perspectives in Iquitos, Peru.

## Methods

### Setting

This study took place in the MRIS network, the largest public health network of Loreto, located in the Peruvian Amazon. This health network has 20,000 women aged 30–49 years who are eligible for cervical cancer screening. The capital city of Loreto is Iquitos (total population 400,000). Iquitos is the largest city in the world without road access; it can only be reached by boat or plane. The main sources of income for this region come from fishing, agriculture, logging, oil extraction, tourism, and small businesses.

The *Seguro Integral de Salud* (SIS) is the largest public health insurance program in Peru for the otherwise uninsured, targeting those living in poverty and extreme poverty. Those with SIS benefit from full or partial subsidization of insurance payments and have access to a network of public primary care and public hospital facilities around the country. In Loreto, 67% of the population is covered by SIS [21]. All health centers in this study were SIS facilities.

The MRIS network is composed of 17 primary care facilities, one of which is a larger health center with its own laboratory, while the rest are smaller health posts staffed primarily by few nurses or nurse-midwives, known as obstetras. The nurse-midwives are the first point of contact for all women’s health services, including cervical cancer screening. These 17 facilities serve populations within a given radius and refer their patients to the larger health center or one of the two regional hospitals in the Iquitos city center, as needed. The furthest primary care facility is approximately 58 km away from the two hospitals in the city, while the closest is about 5 km away. Within Iquitos city, the main mode of transportation is motorcycle taxi. There is one paved ~ 100 km stretch of highway along which many MRIS health facilities are located. However, on the many unpaved roads in Iquitos or for women living in communities along the rivers, it can take hours to reach a larger health center or one of the regional hospitals by motorcycle taxi or a combination of boat, foot, bus, and motorcycle-taxi.

According to MINSA's national cervical cancer prevention and control plan for 2017–2021 (referred to as ‘the MINSA plan’ hereafter), women 30–64 years of age should undergo annual cervical cancer screening. Women 30–49 years old should have an HPV test or VIA test while women 50–64 years old or who are pregnant should have a Papanicolaou (Pap) smear test. The MINSA plan also recommends implementation of a screen-and-treat program in which women with positive HPV or VIA results are offered ablative treatment (if eligible) in the same location as the screening, without need for referral. If the screen-positive woman is not eligible for ablative treatment (e.g., due to lack of visibility of the transformation zone of the cervix), she should then be referred for colposcopy at the hospital level. Women with a positive Pap should always be referred for colposcopy. Following the colposcopy, women are recommended a treatment appropriate to the severity of their infection and followed up with until they complete the recommended treatment [5].

However, despite these recommendations by the MINSA plan, in the MRIS network, there was no capacity to offer HPV testing or ablative treatment at the primary care facilities until late 2019. The primary care facilities offered only VIA and Pap screening tests and, in some cases, would screen women with both tests. Similarly, though the MINSA plan for ablative therapy at the primary level was recommended for VIA or HPV positive women, all resources (medical equipment and trained staff) required for ablative treatment were only available at the two regional hospitals. All other forms of treatment (excisional or otherwise) and colposcopy could also only take place at the two hospitals. Based on investigations of existing capacity conducted by Proyecto Precancer, at any given time between 2017–2019, an estimated five doctors across the two hospitals were performing colposcopy and ablative/excisional treatments during limited time slots, and each hospital only had one set of equipment for these procedures.

This meant that all screen-positive women from the MRIS network would have needed to be referred to a hospital for colposcopy until late 2019, including the participants in this study. The hospital referral is given to screen-positive women when they return to the primary care facility to receive their result or counseling about their result. There is no standardized system for telling women to return to the primary care facility for their referral; some women are told to return at the end of their screening procedure to receive their result and referral, if needed, and others receive a phone call or house visit telling them to return to the primary care facility for their referral. When women receive their referral, they also receive a referral form, which is required in order to seek care at the hospital level. At the time of this study in 2019, this referral form was only valid for two months. If the referral form expired before the continuum of care was completed, women needed to return to primary care for another form.

**Fig. 1:**
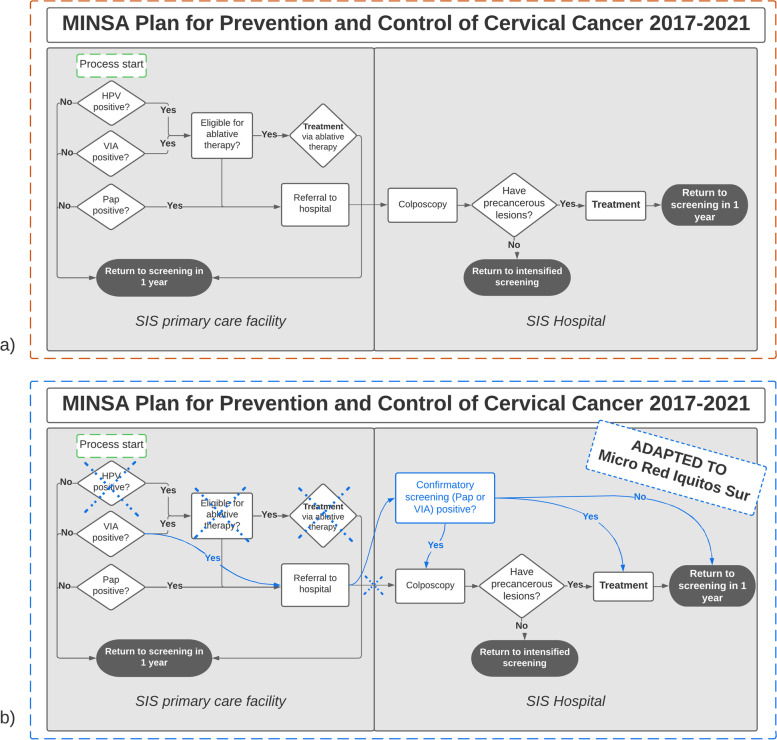
The Screening and Treatment Continuum of Care. Note. **a** depicts the screening and treatment continuum of care according to the MINSA’s national plan for prevention and control of cervical cancer 2017–2021. **b** shows how the MRIS network has adapted the MINSA plan in accordance with existing resources (in blue)

Care at the hospital level also deviated from the MINSA plan; in many cases, screen-positive women who were referred to the hospital were screened a second time at the hospital to confirm their abnormal result prior to colposcopy. If the result of the confirmatory screen was negative, they were referred back to primary care for routine screening in one year (see Fig. 1).

The two hospitals use a patient-level information system containing information for every appointment and follow-up procedure. This system and the primary-level system are used to inform the MINSA plan’s indicators (i.e., percentage of women 30–49 years old screened with VIA, percentage of screen-positive women who received treatment). All cervical cancer screening is performed at the primary care centers, and all follow-up for women with abnormal results is completed at one of the two hospitals. The primary health care centers have patient’s information regarding the cervical cancer screening results, and the hospitals have the information regarding appointments and/or follow-up procedures. However, these information systems are not interconnected, and there is no systematic follow-up of patients to confirm whether they have completed their care. Therefore, if a nurse-midwife from a primary care center wishes to confirm if a specific referred patient has completed her care, they must personally contact the hospitals.

### Participant selection and procedures

We used purposive sampling to select participants. All women who were eligible to participate in this study had an abnormal screening result recorded at one of the 17 primary care SIS facilities in the MRIS network between January 2018 and February 2019 and had no record of having reached an ‘endpoint’ in the continuum of care in either of the two SIS hospitals. An endpoint could be either (1) receiving a negative confirmatory screening result or (2) completing treatment.

According to routinely collected monitoring and evaluation data, 503 women screened positive with either Pap or VIA between January 2018 and February 2019. To identify screen-positive women from this group who had not received follow-up, the project team first worked with primary care nurse-midwives to identify women who had been referred to hospital following an abnormal screening test result. We then used the hospital-level information system to cross reference each of the screen positive women and verify whether they reached an endpoint. Any woman for whom there was no record of having reached an endpoint was considered lost to follow-up and was eligible to participate in this study.

The aim of recruitment was to identify 20 women who were lost to follow-up. Once 20 women were identified, the nurse-midwives first called each woman, and if they agreed to be interviewed, they were contacted by the project team over the phone (if they owned one) or by a house visit to coordinate the interview. All 20 women agreed to be interviewed. Interviews were conducted in June 2019 by a female, Peruvian project collaborator (L.E.C.J.) with a Master’s in Public Health and experience working in cancer prevention in Iquitos. The interviews were semi-structured and discussed topics including emotions related to positive screening results, challenges associated with receiving follow-up care, alternative treatments (e.g., treatment external to the SIS system), and suggested changes to improve the system. All interviews were conducted in Spanish and took place in participants’ homes. Interviews were recorded and transcribed. Field notes were taken and used to create greater understanding of the interviews but not used for data analysis. No additional women were recruited for the study beyond the initial 20 women due to saturation; the research team observed repetition in the barriers faced by women and no new findings emerging from the data.

### Ethics approval

The study was reviewed and approved by all participating ethical institutional review boards at Asociación Benéfica PRISMA (CE0251.09), Tulane University School of Public Health and Tropical Medicine (reference number 891039), the University of Maryland School of Medicine (IRB#061,614), Hospital Regional Loreto (ID-002-CIEI-2017), and Hospital Apoyo Iquitos (065-ID-ETHICS COMMITTEE HICGG- 2018). Written consent was obtained prior to the interviews.

### Coding and analysis

Two researchers (M.J. and J.B.) used an inductive thematic analysis approach to analyze the interview transcripts. They first developed a codebook and double-coded all transcripts using Dedoose [22]. Intercoder reliability was checked with this program. Any coding differences were discussed between the coders and resolved. Minor additions and edits were made to the codebook during the coding process. Researchers then reviewed all transcripts to ensure they were coded in line with the final version of the codebook.

The Health Care Access Barriers Model is a framework for classifying, analyzing and reporting measurable and modifiable health determinants. It also serves as a practical tool for root-cause analysis, making it an appropriate frame of reference to structure the thematic analysis [23]. To report our findings, we retrospectively grouped our codes across three categories of underlying causes of health disparities, as defined by this model: cognitive, structural, and financial.

## Results

**Fig. 2:**
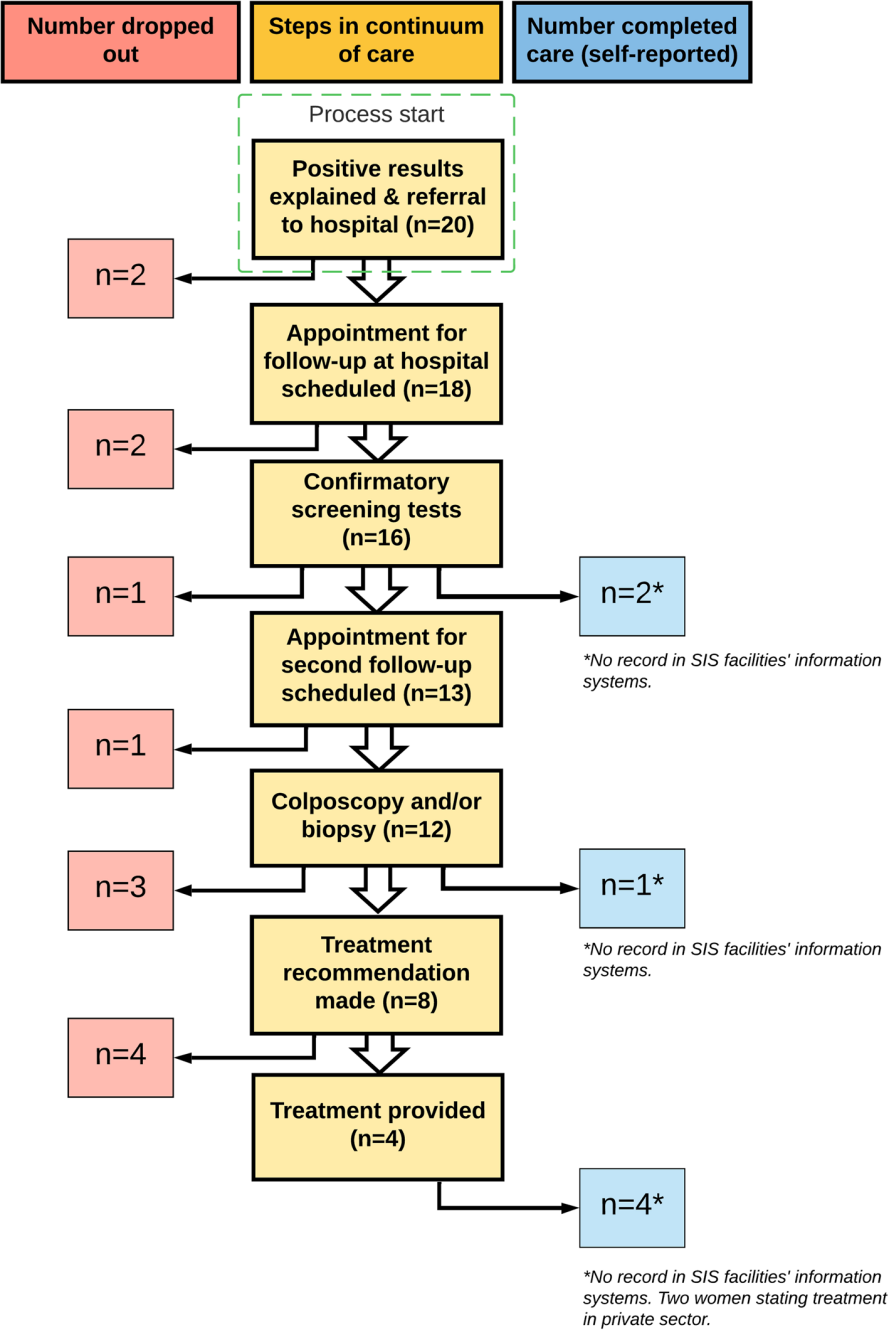
Where women were lost-to-follow-up or completed care in the continuum of care. Note. The yellow boxes depict the steps in the continuum of care while the red boxes show how many women dropped out a given step, and the blue boxes show how many women completed care at a particular step in the continuum of care

In total, twenty women for whom there was no record of reaching an endpoint of care following a positive screening test were interviewed (average age 48 years). Seven women reported, during their interviews, that they did reach an endpoint, despite there being no record of this: three received negative confirmatory test results and four underwent treatment. Two of the women who reported having undergone treatment paid for their treatment in the private sector. These seven women were not excluded from the study as their stories elucidated barriers to follow-up after a positive screening result, as described in the following section. Figure 2 summarizes what happened to each of the twenty women interviewed.

### Main barriers to completing care in the SIS public health system

We summarize our main themes in Table 1 before discussing them further in the sections below.

**Table 1:** Main themes regarding completion of the cervical cancer screening continuum of care

Themes identified	
Enablers	High motivation to complete the continuum of care
	Providers facilitating creative work-arounds for improved access to care
Cognitive barriers	Lack of awareness of HPV/cervical cancer
	Lack of understanding of screening, follow-up and treatment procedures
	Poor communication by healthcare staff
	Patients’ anticipation of challenges with seeking follow-up care
Structural barriers	Challenges with scheduling appointments
	Unavailability of providers
	Long wait times
	Multi-step care processes
	Equipment unavailability
Financial barriers	Out-of-pocket payments
	Costs related to travel or missing days of work

All of the women interviewed showed a strong desire for follow-up care. One woman stated after receiving her positive screening result, “*As soon as I found out, I went and made my appointment,”* (*Participant #1, 31–35 years)* while two others stated:

*If they tell me I have to leave tomorrow and at night, I don't know what I have to do, but I will go. By car, walking, I don't know, I will get up early, but the thing is that I will go (Participant #10, 31-35 years).*

*“With these results we are going to refer you to the hospital in Iquitos, but you are going to go," he [the nurse-midwife] told me. "You are not going to stop going.” "Yes," I told him. "If possible, I will go tomorrow," because it was already one o'clock, more or less, when I went to pick up my results. "Tomorrow I will go early," I told him. And I went (Participant #3, 56-60 years).*

The number of trips for follow-up diagnostic triage or care ranged between two and twelve, with an average of five attempts to complete the continuum of care. The seven women who self-reported having reached completing care, made an average of seven attempts to complete care.

### Cognitive Barriers

Four main cognitive barriers emerged: (1) lack of awareness of HPV/cervical cancer, (2) lack of understanding of screening, follow-up and treatment procedures, (3) poor communication by healthcare staff, and (4) patients’ anticipation of challenges with seeking follow-up care.

Women reported that their providers often mentioned cervical cancer when explaining the meaning of a positive screening result. Many women linked their positive result with a cancer diagnosis: *“In the beginning, I was shocked when the nurse-midwife told me I had cancerous cells” (Participant #18, 41–45 years).* Seven women specifically expressed fear and concern that their positive screening result was a terminal diagnosis:

*It shocks you, just scares you, you know? When they told me ‘I’m in the beginning stages of cancer’, I got scared… I thought of my kids… sometimes you see people who die. I just thought… ‘I can’t be like that’ (Participant #12, 36-40 years).*

Women lacked an understanding of results which was compounded by incomplete explanations of the purpose of a screening test: *“To me, in [the health post], they did the Papanicolaou test, and it showed that I have… a normal urinary tract infection” (Participant #11, 31–35 years).*

Many women described feeling confused about the different screening and follow-up tests, as well as the meaning of confirmatory or diagnostic results:

*They are doctors, and they do not explain it well to us. I don’t understand them… we ask questions, and they need to give us an answer. She didn’t give me an answer. It’s no good (Participant #17, 66-70 years).*

*When you’re there, they don’t explain anything. You enter, and if you say you expect to have a colposcopy done, they don’t explain how the procedure is for a colposcopy; they don’t tell you what they are going to do (Participant #10, 31-35 years).*

Eight women described confusion regarding the numerous procedures required of them, and sometimes this confusion was due to being asked to repeat procedures:

*They screened me so many times. The nurse-midwife did [a physical exam as part of a screening/diagnosis] to me twice, then the doctor, then I went to the hospital, and they did it four more times… (Participant #9, age not reported).*

Poor communication between providers and discordant recommendations given to patients discouraged women from completing follow-up. One woman described the uncertainty and confusion after having received disjointed recommendations from multiple doctors:

*No one would give me a clear result. One said this, another said that… I didn’t know what to do. [I decided it would be] best if I wait the seven months to pass like the one doctor said (Participant #1, 31-35 years)*.

Despite wanting to, two women did not attempt to seek follow-up care as they anticipated that the process would be costly and complicated based on prior experiences:

*If they send me to [either hospital], that’s going to take time, right? I will also have to spend money. It’s not just what the insurance covers. You also have to get there (Participant #12, 36-40 years).*

You know, to go there it is a lot of paperwork, they make you wait… Besides, my child had an accident, and I have more urgent matters to tend to (Participant #13, 46–50 years).

### Structural Barriers

Five main themes emerged within structural barriers in the system: (1) difficulties with scheduling appointments, (2) provider unavailability, (3) long wait times, (4) multi-step care processes, and (5) equipment unavailability.

Most women reported having to travel to their primary care facility just to make an appointment to receive their screening result. One respondent expressed frustration with this:

*“I went [to pick up results]... first, I went to make an appointment. They told me appointments were full, that I have to call. The next day I called, but in the [health] post, they don’t answer when you call” (Participant #12, 36-40 years).*

Eight women reported never being informed of their screening test results. Instead, they were referred to the hospital without an explanation, contributing to the cognitive barrier of poor understanding of the process:

*“They sent me to [the hospital], they gave me a referral. But they never gave me my Pap result”* (*Participant #2, 46-50 years*).

At the hospitals, appointments must be scheduled in person with administrative staff, and several documents are required. These requirements are not always communicated. Two women recounted making the trip to the hospital to make an appointment and were turned away due to missing documents.

*I went the next day, and when I arrived, they asked for a copy of my ID and referral form. “I didn’t bring it,” I told them. “You have to bring it. How can we help you if you don’t bring it?” “Ah, ok. Tomorrow I will come,” I told her, and I did not go back again (Participant #16, 61-65 years).*

*There were no doctors available. I asked, “Ma’am, until when? My referral is going to expire.” “Yes, well, there are [no appointments].” They were on strike… they were even saying on the radio they were on strike (Participant #2, 46-50 years)*.

It was common to make multiple, unsuccessful attempts to receive follow-up care at the hospital. Seven women reported going in to schedule an appointment and being asked to return another time because no appointments were available:

*There are no [appointments]. They called the doctor, and they don’t know if there will be any this month or the next. They really don’t know. Really, when there’s no doctor, who will do the biopsy or the colposcopy? (Participant #2, 46-50 years)*

Five women who had a confirmatory screening test at the hospital never received the results. They were frustrated to learn that the results had been “lost” and that they would need to do another screening test. Additionally, it was challenging for women to make repeated trips to pick up results or redo screening tests at their own expense.

Providers' unavailability at the hospitals on scheduled appointment days was also common. Half [10] of the interviewed women reported showing up for their scheduled appointment only to find out that the health provider they were scheduled to see had already left for the day or was absent.

*Sometimes I wasn’t seen at the hospital when I went for my appointment. Sometimes the doctor left after I already paid for my consultation, so I had to pay again for another visit on another date. That’s how it was (Participant #11, 31-35 years).*

In many cases, seeking care at the hospital requires devoting an entire day due to long wait times. Six women described arriving at their appointment, only to find multiple women were also scheduled for the same time:

*They scheduled me for one o'clock… At one o’clock, the doctor didn’t arrive; at 4 or 5 they would arrive. That’s when I was seen. I get home at 6:30. It’s dark. But I never gave up because it’s my health (Participant #19, 51-65 years).*

*I had gone at 6 in the morning, and I left there at 7 at night… I was so angry because… what’s going on? Why am I here? Why won’t they see me? (Participant #17, 66–70 years)*.

Two interviewed women were recommended a treatment but were unable to receive it because the necessary equipment was not working:

*I returned to the doctor, and he said, “yes, I remember you.” I told him I wanted to do my LEEP cone treatment. And he says, alright, give me your phone number, because right now the equipment is broken. So, I returned a second time… and the same doctor says “equipment is still broken” he told me. The third time I went, he said the same, so I didn’t tell him anything anymore. I stopped going (Participant #18, 41-45 years).*

### Financial Barriers

Fourteen of the twenty women interviewed mentioned direct or indirect costs as barriers to care. Direct costs were having to make out of pocket payments for medicine, supplies, or procedures, and indirect costs were related to travel costs or opportunity costs as a result of missing multiple days of work.

Even though the SIS is designed to protect against out-of-pocket payments, seven women reported making out-of-pocket payments directly related to their care. In some cases, women were asked to undergo additional tests to inform their treatment recommendation. Not all of these tests were covered by SIS. Out of pocket payments were also made for materials necessary for procedures, such as latex gloves.

*I was getting care through SIS. Well, all the tests they could do with SIS, they did. But for what I could not get covered by SIS, they sent me to private care, and privately, I had two tests I had to do that cost me 130 [soles] for both… Then they gave me another order for a pelvic ultrasound, and they asked me to buy two things for that ultrasound which cost 105 [soles] each [Total of* ~*US$130] (Participant #16, 61-65 years).*

*I spent money on travel. Sometimes they made me buy gloves. I had to pay to get tests done. I have to buy things at the pharmacy… 21 or 18 soles [US$6–7] each time (Participant #19, 46–50 years)*.

Women also often mentioned cost of travel to get to the health facilities as a barrier to care:

*I went on the 30th [to pick up my result], [the doctors] were in a meeting, and did not see patients. I returned home. Yesterday, I didn’t go again because I didn’t have money to get there (Participant #3, 56-60 years).*

*I spent it [money] on getting to the hospital and didn’t have any left to go back (Participant #4, 46–50 years)*.

### Consequence of Barriers

The high number of cognitive, structural, and financial barriers experienced throughout the continuum of care at SIS facilities led a number of women to seek care elsewhere, either through traditional herbal remedies or in the private sector. Nine women mentioned seeking or using herbal remedies, and eight of these only did so in addition to seeking medical care or only *after* continuous delays and barriers associated with getting follow-up care at the hospital level:

*Many women say, “if I have this, I am going to leave [Western] medicine, and I am going to get cured with plants, because I’ll take my plants, and they are not going to make me wait so long like they do for you to find out results”… it’s not a matter of one month. It took me almost 6 months of coming and going (Participant #10, 31-35 years).*

The one woman who opted for herbal remedies instead of medical care faced cognitive barriers related to understanding the excisional treatment she was recommended, referring to the procedure as getting “emptied out” – a term used to refer to hysterectomies which are sometimes done:

*When the doctor told me they are going to empty me out because of the tumor, which could be cancerous… well, I don’t want that. I don’t want them to empty me out. I know which [plant] medicines are going to cure me, and this tumor will dry out, and I will be cured (Participant #16, 61-65 years).*

Five women mentioned being recommended to seek care in the private sector, and, in one case, this was even suggested by a primary care professional in the public health system. One woman reported getting treatment privately “*because I wasn’t being given care [in the public system]. Privately, everything is quick, but you have to pay. But in the [public] hospital, you don’t pay, but it takes long*” *(Participant #18, 41–45 years)*.

### Enablers of follow-up care

Although the scope of this study – understanding challenge with receiving follow-up care – does not lend itself to highlighting what works well in the existing EDT system, multiple women spoke highly of providers who showed them kindness and empathy and who did their best to ensure they got follow-up care:

“*[The primary care nurse-midwife] told me… to go to the hospital and look for the nurse-midwife [name given]. She sent me to see her, and I found her, and she saw me immediately” (Participant #6, 46-50 years).*

One woman described how she was able to get treatment due to one midwife who went above and beyond to make sure her patients were well-cared for:

*With [name of hospital midwife], when the results arrived, she would call us… And there she was, we didn’t have to spend money on multiple trips… If that nurse-midwife wasn’t there, I would not have had my LEEP cone treatment. I was so scared, but she made me calm. She talked to me. She prepared me… (Participant #20, age not reported).*

## Discussion

All twenty screen-positive women included in this study expressed a strong desire to receive treatment, with nineteen women seeking treatment from either the public or private healthcare system and one woman resorting to traditional herbal remedies. On average, the women made five attempts to complete the continuum of care. During the interviews, we discovered that seven of the women did complete the continuum of care, despite there being no record of this at the SIS facilities. The disjointed information systems that do not link primary care with hospital care at the patient level put the onus of follow-up on the women. This, combined with the cognitive, structural and financial barriers, makes it extremely difficult for women to receive the care they need.

The cognitive, structural and financial barriers identified in this study reflect health system shortcomings that are common in low- and middle-income settings [11–18]. Our results regarding cognitive barriers are in line with previous studies in Latin America which found that lack of knowledge about cervical cancer screening, follow-up and treatment [11, 14, 15], and poor communication by healthcare staff were barriers to completing the continuum of care following a positive cervical cancer screening result [11, 14]. Similarly, the structural barriers outlined in our study, including delays in the continuum of care, provider and equipment unavailability, and multi-step care processes, were commonly reported as barriers to receiving follow-up care after a positive cervical cancer screening result in Latin America [11–15]. With financial barriers, our study corroborates previous studies in Latin America which found payments during the treatment process, travel costs, and missed opportunity costs from missing work to be barriers to completing the continuum of care following a positive cervical cancer screening result [12–14, 17, 20].

The consequences of facing barriers to follow-up care included turning to traditional herbal remedies. Use of traditional herbal remedies is common practice in Iquitos, and this study found that the herbal remedies are mainly used in conjunction with medical care, after numerous attempts to receive care through the public health system, or due to fear associated with a poor understanding of the treatment recommended by healthcare professionals. Nevin et al. [3] similarly found that traditional herbal remedies were used by Peruvian women when the barriers to receiving follow-up care were insurmountable.

These many barriers can be further understood from the perspective of Complex Systems Science, which studies how systems that are comprised of multiple, interacting components self-organize and adapt in response to internal constraints or pressures [24]. In the MRIS network – a complex system that consists of the primary and hospital levels of care – the reality of the EDT program shows that the local health system had to ‘self-organize’ in accordance with the resources available rather than following the recommended template of the MINSA plan. For example, one adaptation was the addition of confirmatory screening, which was implemented in good faith to ensure that only those who need a colposcopy are scheduled for one; however, it led to unanticipated consequences, such as women becoming discouraged by the repeat clinical exams and screening tests, causing them to drop out before getting treatment. Moreover, there is a complex interplay between cognitive, structural, and financial barriers where each barrier compounds the impact of the other. For example, the structural barrier of healthcare providers being unavailable when women show up for their scheduled appointments leads to an increased financial barrier as women must pay for transport to the hospital again.

While many strategies have been evaluated for improving management of cervical precancer and cancer [25–30], they are largely not feasible for improving rates of loss to follow up in contexts such as the MRIS network. For example, in low-income settings, cryotherapy is commonly used for treatment of cervical cancer [31]. However, implementing cryotherapy in the MRIS requires transporting bulky gas containers to remote locations, which then have to be shipped back to Lima, Peru for refilling. This cumbersome process renders the use of cryotherapy largely impractical as a cervical precancer and cancer management strategy in the MRIS. Instead, because the screening and treatment continuum spans multiple levels of care, an integrated solution with input from a multidisciplinary group of stakeholders is needed. One way to do this is through workshops with key stakeholders. The Proyecto Precancer study team conducted a series of workshops with key stakeholders, which resulted in agreement to optimize and improve the EDT program through implementation of a primary-level screen-and-treat approach with HPV testing followed by visual triage and ablative therapy for eligible women. This approach is supported in the WHO’s 2013 guidelines on cervical cancer early detection and treatment programs [32]. Introducing a treatment option at the primary health facility level circumvents the barriers related to accessing hospital care and increases the hospitals’ capacity to provide more timely care to those who really need specialist care by reducing the number of referrals. Our monitoring and evaluation of the MRIS network revealed that after the introduction of this screen-and-treat program in 2019, the proportion of women who were lost to follow-up decreased from 69.8% to 30.0% [20].

While this is a significant step forward, implementation of more advanced screening and treatment technologies will not single-handedly solve the problem of loss to follow-up. Even in the screen-and-treat approach, some women will be ineligible for ablative therapy at the primary level and will need a hospital referral.

A more complete and integrated information system that allows communications across levels of care could reduce the number of trips these women need to make if the primary care professional were able to schedule their hospital appointment immediately upon determining ineligibility for ablative therapy. On the condition that personal health data is appropriately protected, an open flow of communication between levels of care could not only streamline referral and counter-referral processes [33], it could also address cognitive barriers by ensuring that primary care professionals who see patients more frequently are kept updated about a woman’s care at the hospital level so that they are able to clarify any misunderstandings at the next point of contact. Additionally, examining and finding solutions to the hospital level barriers would benefit other patients accessing these services, whether by improving the appointment system process or streamlining hospital level procedures.

Cognitive barriers could be addressed more directly via the development of specific guidelines and tools to support professionals with explaining screening results and treatment options in a way that is locally adapted and culturally appropriate. Such guidelines would ensure standardization and consistency of key messages given to women and reduce the likelihood of misinterpretation and confusion [34].

The barriers discussed in this study demonstrate the complexity of implementing cervical cancer EDT programs and show the value of incorporating women’s perspectives to understand the complex system and identify areas to leverage change for improving follow-up among screen-positive women. In the context of the fifth Sustainable Development Goal of achieving gender equality, these findings represent an opportunity for regional governments to improve systems that will reduce loss to follow-up and in turn reduce the rate of preventable mortality due to cervical cancer [35]. Making improvements in the information systems, multidisciplinary working across levels of care, and patient communication will address many of the barriers that lead to loss to follow-up in low-and-middle income settings.

### Limitations

Although we interviewed women for whom there was no record of having completed follow-up to understand their experiences, we note that a lack of *documented* completion of care is not always indicative of incomplete care, as shown in our interviews. However, all women’s experiences in this study elucidated challenges with the system. As is the case with most qualitative studies, these findings are not representative of the entire population of the region nor are they generalizable to other regions of Peru. However, the themes that emerged provide valuable information about the barriers women face. Themes that emerged were repetitive and consistent among the women. Moreover, to address limitations of qualitative research, the interviews in this study were completed by a local researcher with experience in cancer prevention in the region. The data were also coded and analyzed by two researchers.

## Conclusion

Women with positive cervical cancer screening results in Iquitos are motivated to complete follow-up care. Despite their best efforts, most women faced a combination of cognitive, structural, and financial barriers as they sought follow-up care. These barriers highlight the complexity of implementing cervical cancer EDT programs, which must take into account perspectives from all stakeholders, including the women, in order to be successful. A linked-up information system that tracks where a woman is in her continuum of care is needed not only to verify what needs to be done next but also to make it easier to follow-up with women who have stopped showing up. Multilevel, coordinated system interventions informed by systems thinking and user-centered design are needed to improve follow-up among screen-positive women [36–38].

## Data Availability

Data and materials are available on request to the corresponding author.

## Abbreviations

HPV: Human papillomavirus
EDT: Early Detection and Treatment
MINSA: The Peruvian Ministry of Health
VIA: Visual inspection with acetic acid
MRIS: Micro-Red Iquitos Sur
SIS: *Seguro Integral de Salud* [Comprehensive Health Insurance]
